# Clinical application of enhanced recovery after surgery concept in laparoscopic treatment of pediatric acute appendicitis

**DOI:** 10.1007/s00383-023-05439-5

**Published:** 2023-04-11

**Authors:** Shao-Min Zhang, Jie Chen, Hui Li, Meng-Fu Guo, Nuan Han, Jin-Song Sun, Chong-Fang Zhang, Lin Su

**Affiliations:** 1 Department of Clinical Medicine, Jining Medical University, N45 Jianshe South Road Rencheng District, Jining, 272013 Shandong Province China; 2 Department of Pediatric Surgery, Affiliated Hospital of Jining Medical University, N89 Guhuai Road, Jining, 272029 Shandong Province China; 3 Department of Hernia and Pediatric Surgery, Quzhou People’s Hospital, Smart New City Minjiang Avenue No. 100, Quzhou, 324000 Zhejiang Province China

**Keywords:** Enhanced recovery after surgery, ERAS, Appendicitis, Pediatric surgery

## Abstract

**Background:**

This study assesses whether enhanced recovery after surgery (ERAS) is beneficial in treating acute appendicitis in pediatrics by laparoscopic techniques.

**Method:**

The children with acute appendicitis (n = 116) were divided into the ERAS group (n = 54) and the control group (n = 62). Then the preoperative data, intraoperative observation indexes, and postoperative data were analyzed.

**Results:**

There was no significant difference in preoperative data and intraoperative observation indexes between the two groups. C-reactive protein (CRP) and white blood cell (WBC) in the ERAS group were significantly lower than those in the control group 3 days after the operation. Moreover, no significant difference in the visual analog score (VAS) between the two groups 3 days after the operation, but the other postoperative observation indexes in the ERAS group were significantly better than those in the control group. Nausea and vomiting in the ERAS group were significantly lower than those in the control group, with no significant difference in other complications between the two groups.

**Conclusion:**

ERAS could improve children’s comfort, reduce some postoperative complications, reduce hospitalization expenses, and speed up recovery from acute appendicitis treated by laparoscopy. Therefore, it has clinical application value.

## Introduction

Acute appendicitis is one of the most common diseases in the acute pediatric abdomen, with approximately four times the incidence in children than in the general population [1]. On the one hand, the development of children's physiological functions and anatomical structure are incomplete, so pediatric appendicitis often has atypical symptoms, rapid disease development, easy perforation, suppuration, diffusion into sepsis, and even toxic shock. On the other hand, children have limited cognition and comprehension ability and often cannot accurately and timely express their disease status. Usually, the appendix has gangrene or perforation, resulting in severe abdominal adhesions, increased difficulty of surgery, postoperative residual abdominal infection, and intestinal obstruction. Therefore, pediatric acute appendicitis should be actively treated once diagnosed, and laparoscopic appendectomy is the preferred surgical method for most pediatric acute appendicitis [2]. This study introduces the concept of ERAS and applies the new surgical concept of ERAS to the laparoscopic treatment of pediatric acute appendicitis, which confirms its application value in pediatric surgery.

## Materials and methods

### Research object

One hundred sixteen children with acute appendicitis who underwent laparoscopic surgery in the Department of Pediatric Surgery, Affiliated Hospital of Jining Medical University, were selected from September 1, 2020, to October 30, 2021. Among them, 54 children were set up in the ERAS group after obtaining the informed consent of their parents; Sixty-two children were treated with routine perioperative management. The above two protocols were administered separately by the same medical and nursing team, and the specific interventions included preoperative interventions (Table 1), intraoperative interventions (Table 2), and postoperative interventions (Table 3). Inclusion Criteria: (1) Age between 6 ~ 14 years old; (2) Symptoms, signs, imaging, and laboratory tests meet the diagnostic criteria for acute appendicitis and are treated with laparoscopic surgery; (3) American Society of Anesthesiologists (ASA) grade I ~ II.

**Table 1:** Comparison of pre-operative measures between the ERAS group and experimental group

ERAS group	Control group
1. Improve abdominal ultrasound, chest radiograph, blood routine, coagulation routine, inflammatory index, liver and kidney function, electrolyte, and other pre-operative routine examinations;	1. Improve abdominal ultrasound, chest radiograph, blood routine, coagulation routine, inflammatory index, liver and kidney function, electrolyte, and other pre-operative routine examinations;
2. Evaluate the children's condition and vital signs in detail, correct electrolyte imbalance and acid–base imbalance in time, and use anti-anaerobic bacteria combined with third-generation cephalosporin antibiotics to control infection;	2. Evaluate the children's condition and control infection with anti-anaerobic bacteria combined with third-generation cephalosporin antibiotics;
3. Detailed pre-operative ERAS education, condition assessment, and introduction, treatment plan, disease prognosis, disease outcome, postoperative rehabilitation, postoperative pain, vomiting conditions, and solutions, expected hospital stay and related appeals;	3. Routine education on the condition introduction, postoperative pain, abdominal distention, vomiting, and other situation solutions, disease outcome characteristics, treatment, rehabilitation plan, etc.;
4. 8 h before surgery, no solid food, 6 h no dairy products, 4 h no breast milk, 2 h before surgery, can drink sugar water or clear drinks (3 ~ 5 mL/kg);	4. 8 h before surgery, 6 h of dairy products, 4 h of water abstaining;
5. The gastric tube is not routinely placed before surgery; if the fasting time is not enough, the gastric tube can be inserted before the operation and removed after the operation;	5. Routine indwelling gastric tube before surgery;
6. Before surgery, the children are instructed to urinate, and the urinary catheter is not routinely placed	6. The children are instructed to urinate before surgery, and the urinary catheter is not routinely placed

**Table 2:** Comparison of intraoperative measures in the ERAS group and experimental group

ERAS group	Control group
1. Intravenous aspiration compound + tracheal intubation general anesthesia;	1. Intravenous aspiration compound + tracheal intubation general anesthesia;
2. Give local infiltration anesthesia of the incision (lidocaine 2%) before cutting the skin and suturing;	2. Closely monitor vital signs to prevent hypothermia;
3. Closely monitor vital signs, use warm blankets to prevent hypothermia, and maintain body temperature ≥ 36 °C;	3. If necessary, normal saline for abdominal irrigation, sterile gauze dipped in pus; irregular placement of drainage tubes
4. If necessary, normal saline for abdominal irrigation, sterile gauze dipped in pus; irregular placement of drainage tubes;	
5. If the gastric tube is indwelled, the gastric tube is removed after the operation

**Table 3:** Comparison of postoperative measures between the ERAS group and experimental group

ERAS group	Control group
1. The children began to try sugar water six hours after surgery or after fully awake and gradually transitioned from a liquid diet and semi-liquid diet to a regular diet without nausea and vomiting abnormalities (transition every 12 h after tolerance until regular diet);	1. After exhausting, remove the gastric tube, test the drinking water without discomfort and gradually transition to a regular diet;
2. Give children Prophylactic oral ibuprofen suspension before they get out of bed; Pain score > 4 points, followed up with an interval of 6 h;	2. Encourage the children to get out of bed and move after the pain improves;
3. Six hours after surgery or after awakening under anesthesia, encourage the children to get out of bed early with the help of parents or nursing staff;	3. Review blood routine + C-reactive protein and abdominal ultrasound three days after surgery and before discharge
4. Review blood routine + C-reactive protein and abdominal ultrasound three days after surgery and before discharge	

### Data collection

General information: age, sex, Body Mass Index **(**BMI), American Society of Anesthesiologists (ASA) score. Preoperative laboratory indicators: body temperature, heart rate (HR), blood pressure, albumin, hemoglobin, white blood cells, C-reactive protein, sodium (Na^+^), potassium (K^+^), chloride (Cl^−^), and bicarbonate (HCO- 3) biochemical indexes. Intraoperative observations: intraoperative blood loss, intraoperative infusion volume, operation time. Postoperative data collection: postoperative pathological classification, WBC and CRP, first exhaust time, time of getting out of bed, pain score, length of hospital stay, hospital cost, the satisfaction of children and parents, nausea and vomiting, incision infection, residual abdominal infection, intestinal obstruction, and readmission.

### Statistical methods

We used SPSS statistical software version 25.0 for statistical analysis. The measurement data did not conform to the normal distribution after the regular test and used the median and quartile [M (P_25_, P_75_)]. The comparison between groups adopted the nonparametric test. The counting data is expressed in frequency and rate using the χ^2^ test. In the chi-square test, use the χ^2^ continuity correction chi-square test if there is a theoretical frequency of 1 ≤ T < 5; use the Fisher exact probability method if there is a theoretical frequency of T < 1. A Logistic regression model was used to perform a multivariate analysis of postoperative complications. In the two-sided test, *P* < 0.05 was statistically significant.

## Results

### Preoperative data

#### General information

This study enrolled a total of 116 children, the ERAS group: 39 males, and 15 females, aged 6–14 years, with a median age of 10 (8, 12) years; the control group: 41 males, and 21 females, age 6–14 years, median age 9 (7.75, 12) years. Compared with the control group, no statistical significance in age, sex, BMI, hospital temperature, HR, systolic blood pressure (SYS), diastolic blood pressure (DIA), and ASA grade in the ERAS group (*P* > 0.05) (Table 4).

**Table 4:** General information

	ERAS group (n = 54)	Control group (n = 62)	*P*
Age (years)	10 (8, 12)	9 (7.75, 12)	0.267
Gender			
Boys	39 (72.2%)	41 (66.1%)	0.479
Girls	15 (27.8%)	21 (33.9%)	
BMI (kg/m^2^)	18.88 (15.66, 20.87)	16.83 (14.79, 21.64)	0.107
The temperature on admission (°C)	36.90 (36.58, 38.10)	36.70 (36.60, 37.65)	0.406
HR (times/min)	109.00 (100.00, 120.25)	109.50 (98.00, 120.00)	0.773
SYS (mmHg)	112.50 (105.50, 122.25)	116.50 (107.00, 125.25)	0.131
DIA (mmHg)	66.50 (61.50, 71.75)	68.00 (60.00, 73.25)	0.514
ASA grade			
I	41 (75.9%)	50 (80.6%)	0.537
II	13 (24.1%)	12 (19.4%)	

Data are median, and quartile [M (P25, P75)] or example number (n, %); The difference in *P* < 0.05 was statistically significant

#### Preoperative laboratory indicators

Between the control and the ERAS groups, the differences were not statistically significant in WBC, CRP, hemoglobin, albumin, platelets, blood sodium (Na^+^), blood potassium (K^+^), Blood chlorine (Cl^−^), and bicarbonate (HCO− 3) (*P* > 0.05) (Table 5).

**Table 5:** Preoperative-related laboratory test indicators

	ERAS group (n = 54)	Control group (n = 62)	*P*
WBC (× 10^9^/L)	13.21 (9.70, 17.76)	13.40 (8.08, 16.38)	0.503
CRP (mg/L)	41.50 (17.47, 53.80)	31.15 (19.59, 48.15)	0.299
Hb (g/L)	130.00 (122.00, 137.00)	134.50 (125.00, 141.00)	0.149
ALB (g/L)	47.45 (46.10, 49.28)	47.50 (45.18, 49.63)	0.859
PLT (× 10^9^/L)	292.00 (241.50, 327.00)	289.00 (257.50, 387.00)	0.260
Na^+^	139.00 (136.00, 141.00)	138.00 (136.00, 140.25)	0.214
K^+^	4.33 (4.07, 4.68)	4.31 (4.14, 4.62)	0.829
Cl^−^	106.00 (103.00, 108.00)	106.00 (104.00, 108.00)	0.223
HCO- 3	19.55 (17.93, 22.25)	19.90 (17.08, 21.98)	0.668

Data are median, and quartile [M (P25, P75)]; The difference in *P* < 0.05 was statistically significant

### Intraoperative observation indicators

Between the control and the ERAS groups, differences in the operation time, intraoperative blood loss, and intraoperative infusion were not statistically significant (*P* > 0.05) (Table 6).

**Table 6:** Intraoperative observation indicators

	ERAS group (n = 54)	Control group (n = 62)	*P*
Surgery time (min)	70.00 (59.75, 87.25)	70.00 (54.75, 89.25)	0.727
Intraoperative bleeding (mL)	3.00 (3.00, 6.00)	5.00 (2.00, 5.00)	0.095
Intraoperative infusion (mL)	200.00 (200.00, 300.00)	275.00 (200.00, 400.00)	0.350

Data are median, and quartile [M (P25, P75)]; The difference in *P* < 0.05 was statistically significant

### Postoperative data

#### Postoperative pathological classification and inflammatory indexes

**Fig. 1:**
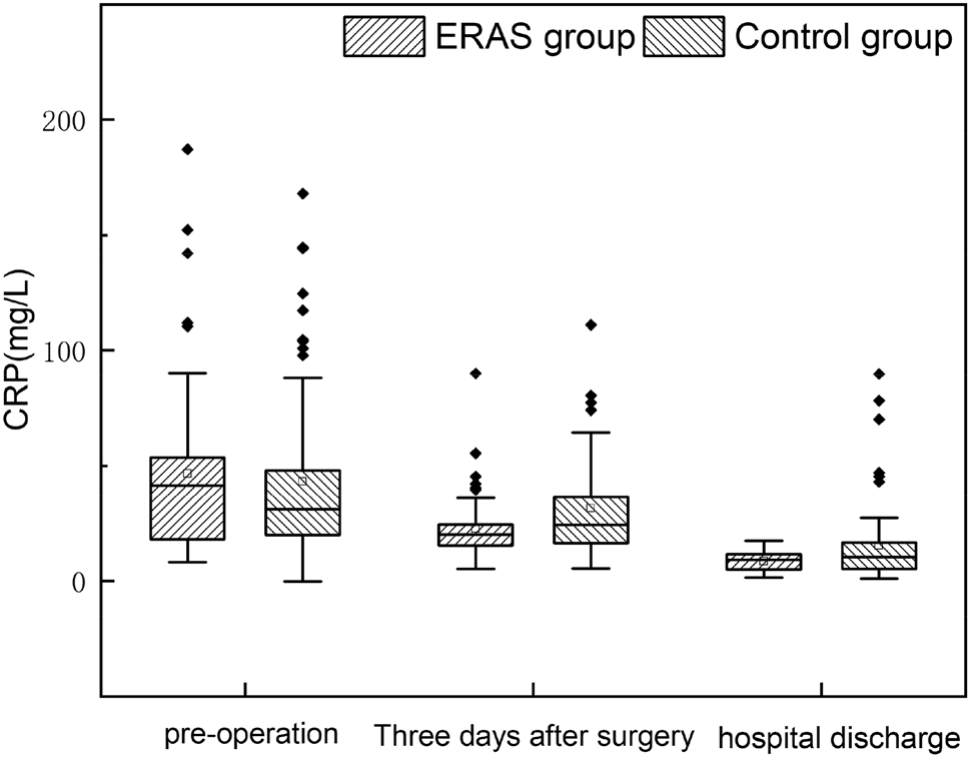
Trend of perioperative CRP index between the two groups

**Fig. 2:**
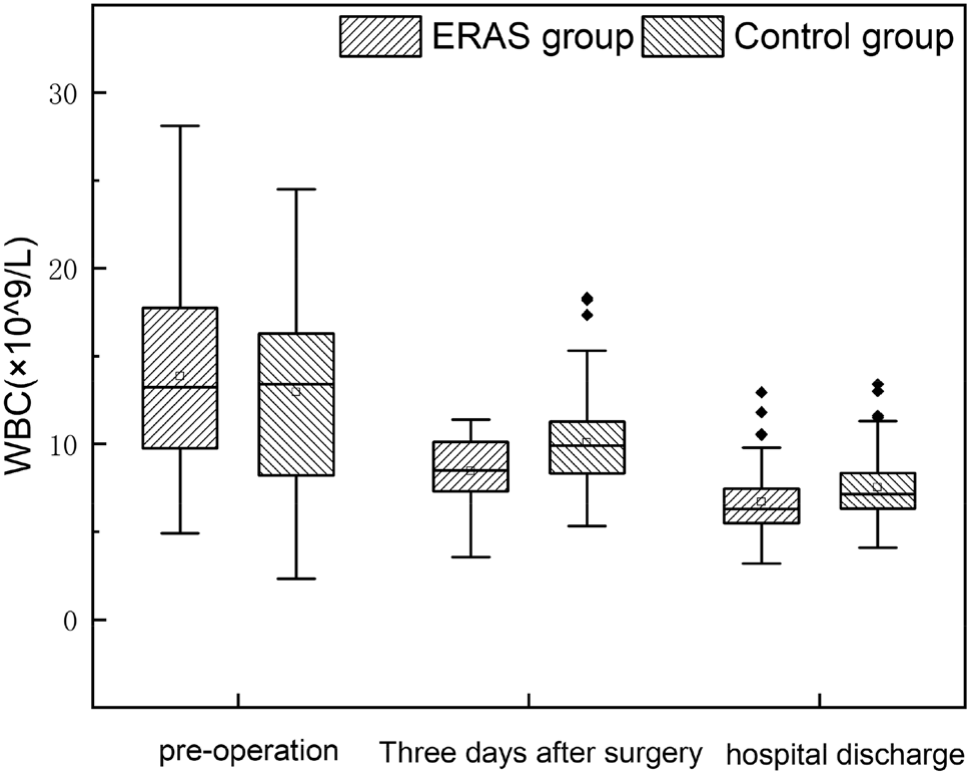
Trend of perioperative WBC index between the two groups

The postoperative pathological classification of the ERAS group: simple 21 cases and purulent 33 cases, control group: simple 25 cases and purulent 37 cases, the two groups were not statistically significant (*P* = 0.875). However, there were significant differences between the groups of CRP 3 days after surgery, WBC 3 days after surgery, and WBC discharge (*P* < 0.05). Moreover, no multiple comparisons of CRP between the two groups (*P* = 0.082) (Table 7). The median change trend of inflammatory indexes in hospital admission, three days after surgery, and discharge of the two groups was the same (Figs. 1 and 2).

**Table 7:** Postoperative pathological classification and inflammatory indexes

	ERAS group (n = 54)	Control group (n = 62)	*P*
Pathotyping			
Simple appendicitis	21 (38.9%)	25 (40.3%)	0.875
Purulent appendicitis	33 (61.1%)	37 (59.7%)	
CRP 3 days after surgery (mg/L)	20.01 (15.27, 24.22)	24.27 (15.95, 36.64)	0.029
Discharge CRP (mg/L)	8.51 (5.02, 11.25)	10.32 (5.34, 16.97)	0.082
WBC 3 days after surgery (× 10^9^/L)	8.45 (7.28, 10.11)	9.92 (8.32, 11.29)	0.006
Discharge WBC (× 10^9^/L)	6.3 (5.48, 7.46)	7.15 (6.32, 8.37)	0.016

Data are median, and quartile [M (P25, P75)] or example numbers (n, %); The difference in *P* < 0.05 was statistically significant

#### Postoperative observation indicators

Between the control and the ERAS groups, the differences were statistically significant in the first exhaust time, first time out of bed activity time, VAS score at 1 day after surgery, VAS score at 2 days after surgery, length of hospital stay after surgery, hospitalization cost, children and parents’ satisfaction (*P* < 0.05). However, there was no statistically significant difference in VAS scores between groups 3 days after surgery (*P* = 0.163) (Table 8).

**Table 8:** Postoperative observation indicators of children

	ERAS group (n = 54)	Control group (n = 62)	*P*
First exhaust time (h)	23.35 (18.52, 28.46)	30.32 (25.89, 36.06)	< 0.001
First exit activity time (h)	9.87 (8.56, 12.35)	17.24 (13.64, 20.34)	< 0.001
VAS score one day postoperatively	8.00 (7.00, 8.00)	8.00 (8.00, 9.00)	< 0.001
VAS score two days after surgery	6.00 (5.00, 6.00)	7.00 (7.00, 8.00)	< 0.001
VAS score three days after surgery	4.00 (4.00, 4.00)	4.00 (4.00, 5.00)	0.163
Length of hospital stay after surgery (h)	106.32 (85.38, 129.61)	159.11 (151.37, 165.42)	< 0.001
Hospitalization expenses (RMB)	11, 881.64 (10, 705.17, 12, 971.82)	13, 700.00 (12, 900.00, 14, 575.00)	< 0.001
Children satisfaction (%)	89.50 (88.00, 94.00)	86.50 (82.75, 89.00)	< 0.001
Parents’ Satisfaction (%)	87.00 (85.00, 89.00)	73.00 (68.00, 78.00)	< 0.001

Data are median, and quartile [M (P25, P75)]; The difference in *P* < 0.05 was statistically significant.

#### Postoperative adverse reactions and complication indicators

**Fig. 3:**
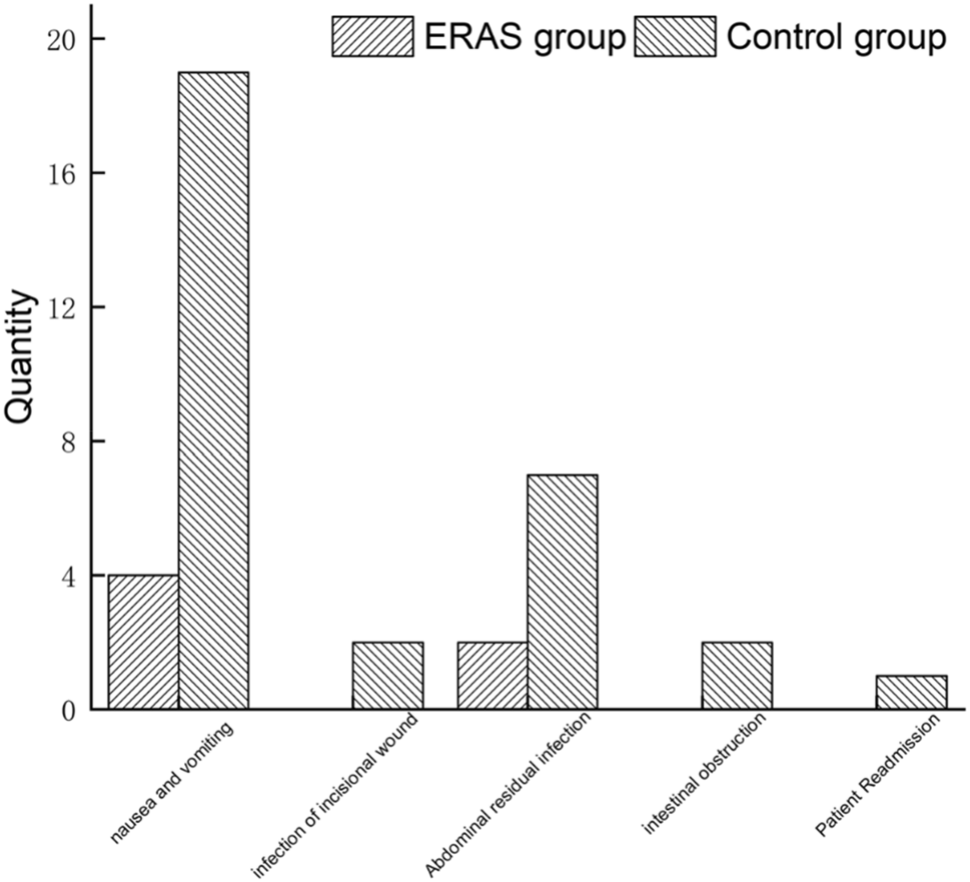
Postoperative adverse reactions and complication between the two groups

The difference between the two groups for postoperative nausea and vomiting was statistically significant (*P* < 0.05). However, there were no statistically significant differences between groups in post-incision infection, residual abdominal infection, hospital readmission, and intestinal obstruction (*P* > 0.05) (Table 9). The number of postoperative adverse reactions and complications in the ERAS group was lower than in the control group (Table 9 and Fig. 3). In a binary logistic regression analysis, the children's postoperative complications were the dependent variable, while all the variables in univariate analysis were independent. No risk factors were identified that significantly influenced the development of postoperative complications.

**Table 9:** Postoperative-related complications

	ERAS group (n = 54)	Control group (n = 62)	*P*
Nausea and vomiting	4 (7.4%)	19 (30.6%)	0.002
Incision infection	0 (0.0%)	2 (3.2%)	0.183
Residual abdominal infection	2 (3.7%)	7(11.3%)	0.128
Ileus	0 (0.0%)	2 (3.2%)	0.183
Readmission to hospital	0 (0.0%)	1 (1.6%)	0.349

## Discussion

Surgeons, such as Henrik Kehlet [3], originally proposed the core concept of ERAS, which integrated multiple high-quality clinical evidence-based medical evidence into perioperative management to improve the perioperative trauma and stress response and accelerate recovery. A 2022 clinical study that applied the ERAS concept to 85 children undergoing surgery showed that the ERAS regimen could accelerate postoperative functional recovery without increasing the risk of postoperative nausea, vomiting, and poor incision healing; However, they mainly discussed pediatric orthopedics [4]. A team from the Hannover Medical School in Germany surveyed the length of stay, patient satisfaction, parent satisfaction, pain management, complications, etc. It recommended that the appropriate ERAS protocol be proposed according to the specific situation of the pediatric patient, which can significantly contribute to the accelerated recovery of children [5]. Ruyue Gao of Zhengzhou University explored the application of ERAS concepts in pediatric gastrointestinal surgery [6]; however, the sample size was small in the trial.

Pain as a fifth vital sign is a bitter experience for almost all perioperative children, but good postoperative pain management is essential in accelerating recovery. Although opioids remain the mainstay of treatment for pain control, their use can have side effects such as suppression of gastrointestinal function, respiratory depression, and postoperative nausea and vomiting, significantly affecting the patient's recovery and prolonging the hospital stay [7–9]. Multi-modal analgesia (MMA), including acetaminophen, nonsteroidal anti-inflammatory drugs (ibuprofen), regional nerve blocks, local infiltrates of incisions, and epidural labor pain, has received much attention in recent years for substantially reducing opioid analgesics [7–13]. In this research, the ERAS group applied local infiltration anesthesia (2% lidocaine) around the incision before skin cutting and suturing and administered an appropriate dose of ibuprofen suspension according to kilogram body weight oral prophylactic analgesia 6 h after surgery or before getting out of bed. When the pain score was more than 4 points, this research gave additional administration after 6 h. By combining multi-modal analgesia, the results of this study showed that the children in the ERAS group had better VAS scores one day and two days after surgery, time of first getting out of bed, postoperative pain, anxiety, and crying, and children and parents’ satisfaction. Good pain management increases the compliance of children with early postoperative activities. And early postoperative getting out of bed can stimulate intestinal peristalsis to a certain extent, promote the recovery of intestinal function, reduce postoperative abdominal distention, and make children more tolerant of early postoperative eating [14]. At the same time, early getting out of bed can also reduce the risk of atelectasis and lung infection and is the simplest and most effective measure to prevent the occurrence of postoperative intestinal adhesions and intestinal obstruction. The average time of the first time out of bed was nearly 6 h earlier than that of the control, which was significantly better than the control (*P* < 0.05) in terms of first exhaust time, postoperative nausea or vomiting, and complications of postoperative intestinal obstruction were not occurring. Non-pharmacological treatments are equally crucial for perioperative analgesia in children. Children who suck lollipops, watch animated videos and games, and are accompanied by their parents are more likely to get out of bed early and tolerate pain better. These measures can shift the focus of pain to other things, thereby achieving analgesia.

The metabolic characteristics of children are different from adults. Glycogen storage capacity and glycolytic activity are relatively weak, so the ability to tolerate hunger is limited. Then malnutrition is more likely to occur after surgery, which affects the incision healing and delays the recovery of children [15]. Therefore, the traditional long-term fasting of water 6 ~ 8 h before surgery will cause hunger, thirst, crying, and restlessness and can even lead to complications such as dehydration, hypoglycemia and insulin resistance, electrolyte imbalance, and shock [16]. The results of this study showed that the first time of eating or drinking and the exhaust time of children in the ERAS group were shorter than those in the control group (*P* < 0.05). Children in the ERAS group adopted the regimen of 8 h before surgery to ban solid food, 6 h to avoid dairy products, 4 h to ban breast milk, and 2 h before surgery to drink sugar water (3 ~ 5 mL/kg). The program effectively alleviated the pre-operative thirst and hunger, reduced the children's crying and restlessness, and improved compliance during diagnosis and treatment. The satisfaction of the children and parents was significantly better than that of the control group (*P* < 0.05). It did not increase complications such as reflux and aspiration compared with the control group, which was similar to the results reported in relevant studies [17–19]. Children in the ERAS group began to try drinking water 6 h after surgery. After no discomfort, such as nausea and vomiting, abdominal distention, etc., they transitioned from clear liquid to a semi-liquid diet within 12 ~ 24 h. They gradually increased the amount and times of eating. The recovery time of intestinal function was significantly better than that of the control. The incidence of complications such as nausea, vomiting, and intestinal obstruction after surgery did not increase [20]. Most children strongly desire to eat in the early postoperative period, and timely early oral eating can significantly relieve the discomfort of hunger, and thirst, improve the postoperative nutritional status of children, and reduce hypoglycemia and insulin resistance.

One of the main goals of the ERAS concept is to reduce the length of hospital stay of children, which has been confirmed by many domestic and international studies and related meta-analyses [3, 16]. The results of this study showed that the median postoperative hospital stay in the ERAS group was about 18 or 19 h earlier than that of the control group, and the hospitalization cost was reduced by 1800 yuan, which enabled the children and parents to return to normal life earlier, and reduced the financial burden of the family to a certain extent.

The traditional concept agrees that a pre-operative prophylactic indwelling nasogastric tube can prevent aspiration after anesthesia and relieve postoperative abdominal distention. However, long-term gastric tube insertion increases the discomfort of nausea, vomiting, and nasopharyngeal pain and delays the children's early eating and getting out of bed. The ERAS group did not routinely place the gastric tube before the operation. If indwelling, extubation after the operation helps children avoid the painful experience caused by the operation of the gastric tube and increases the children's compliance to get out of bed early. In this study, children in the ERAS group had significantly less postoperative nausea and vomiting than the control group, which may be because it avoids reflexive vomiting caused by indwelling gastric tube stimulation of the throat.

CRP and WBC are specific indicators of early infection and inflammation and can also reflect the traumatic stress response of surgery [21]. This study showed that CRP, WBC, and discharge CRP were lower than those in the control group 3 days after surgery (*P* < 0.05). The incidence of hospital readmission and complications, such as residual abdominal infection, incision infection, and intestinal obstruction, was lower than that of the control group, which to a certain extent, indicated that the ERAS program did not increase the occurrence of the above difficulties. Its application in pediatric AA was feasible and safe.

The realization of ERAS results from the promotion of various measures, so selecting appropriate, safe, and effective perioperative measures is essential. Although the ERAS concept just chose part of the elements, the results show that it does accelerate recovery, relieve perioperative pain, and shorten the length of hospital stay. These advantages make us believe that more safe and efficient factors added to the perioperative management of pediatric AA will produce more beneficial changes. Similarly, a multidisciplinary collaboration among clinical staff is necessary for the design and implementation of ERAS programs. Surgeons and anesthesiologists jointly develop standardized postoperative analgesia models. Also, nursing staff and surgeons discuss relevant postoperative nursing measures and emergency treatment of related complications to realize a close connection between all aspects of perioperative care. At the same time, it increases the sense of identity and active cooperation of the children's families. All parties implement the concept of accelerated rehabilitation throughout the process, achieve information exchange and complete cooperation, and implement perioperative management programs centered on the high-quality rehabilitation of patients.

In summary, the ERAS program can reduce postoperative pain, shorten hospital stay, reduce hospitalization costs, enhance children and parents’ satisfaction, and accelerate postoperative functional recovery without increasing postoperative complications. Therefore, it is safe, effective, and feasible to introduce the concept of ERAS into the perioperative care model of laparoscopic treatment of pediatric acute appendicitis.

Shortcomings of this study: Some of the results were not statistically significant; for example, the two groups of children who were discharged from the hospital with CRP (*P* = 0.082) and the 3-day postoperative VAS score (*P* = 0.163) were not statistically significant, which may be due to the limited sample size of this study, and these results require multi-center, large-scale studies to explore further.

## Data Availability

The datasets used and/or analyzed during the current study are available from the corresponding author on reasonable request.
